# Mechanical Restraint in Inpatient Psychiatric Unit: Prevalence and Associated Clinical Variables

**DOI:** 10.3390/medicina59101847

**Published:** 2023-10-17

**Authors:** Andrea Aguglia, Giovanni Pietro Corsini, Isabella Berardelli, Andrea Berti, Benedetta Conio, Nicolò Garbarino, Giovanni Battista Gnecco, Caterina Magni, Enrico Venturini, Alessandra Costanza, Andrea Amerio, Mario Amore, Gianluca Serafini

**Affiliations:** 1 Department of Neuroscience, Rehabilitation, Ophthalmology, Genetics, Maternal and Child Health, Section of Psychiatry, University of Genoa, 16132 Genoa, Italy; andrea.aguglia@unige.it (A.A.); aberti285@gmail.com (A.B.); garbarino.nicolo@gmail.com (N.G.); giovannibattistagnecco@gmail.com (G.B.G.); magni.caterina@libero.it (C.M.); venturinienrico.226@gmail.com (E.V.); andrea.amerio@unige.it (A.A.); mario.amore@unige.it (M.A.); gianluca.serafini@unige.it (G.S.); 2 Istituto di Ricovero e Cura a Carattere Scientifico (IRCCS), Ospedale Policlinico San Martino, 16132 Genoa, Italy; giovannipietro.corsini@hsanmartino.it (G.P.C.); benedetta.conio@hsanmartino.it (B.C.); 3 Department of Neurosciences, Mental Health and Sensory Organs, Faculty of Medicine and Psychology, Suicide Prevention Centre, Sant’Andrea Hospital, Sapienza University of Rome, 00189 Rome, Italy; isabella.berardelli@uniroma1.it; 4 Department of Psychiatry, Adult Psychiatry Service, University Hospitals of Geneva (HUG), 1207 Geneva, Switzerland; 5 Department of Psychiatry, Faculty of Biomedical Sciences, University of Italian Switzerland (USI), 6900 Lugano, Switzerland; 6 Department of Psychiatry, Faculty of Medicine, University of Geneva (UNIGE), 1211 Geneva, Switzerland

**Keywords:** seclusion and restraint, emergency psychiatry, aggression, involuntary admissions, substance abuse

## Abstract

*Background and Objectives*: mechanical restraint (MR) is a controversial issue in emergency psychiatry and should be better studied to implement other alternative therapeutic interventions. The aim of this study was to estimate the prevalence of MR in an Italian psychiatric unit and identify the sociodemographic and clinical characteristics as well as the pharmacological pattern associated with MR. *Materials and Methods*: all subjects (N = 799) consecutively admitted to an Italian psychiatric inpatient unit were recruited. Several sociodemographic and clinical characteristics were recorded. *Results*: The prevalence of MR was 14.1%. Males, a younger age, and a single and migrant status were associated with the MR phenomenon. MR was more prevalent in patients affected by other diagnoses and comorbid illicit substance use, in patients with aggressive behaviors, and those that were involuntary admitted, leading significantly to hospitalization over 21 days. Furthermore, the patients that underwent MR were taking a lower number of psychiatric medications. *Conclusions*: Unfortunately, MR is still used in emergency psychiatry. Future research should focus on the dynamics of MR development in psychiatry, specifically considering ward- and staff-related factors that could help identify a more precise prevention and alternative intervention strategies.

## 1. Introduction

Restraint is a controversial theme of worldwide relevance in psychiatry. It is defined as a limitation in an individual’s freedom of movement, being referred more precisely as a coercive act. The Italian National Bioethics Committee has described restraint as a “mechanical or pharmacological limitation of an individual’s possibility of autonomous movement” [1].

Several types of restraint can be identified, including environmental restraint, defined as a practice or intervention that restricts a patient’s free movement into the psychiatric ward [2]; chemical restraint, consisting in the administration of medication to manage patients’ behavior [3]; and psychological restraint, defined as any decision or program designed to retain a patient’s privileges and participation in activities [4]. Moreover, physical restraint is divided into manual, mechanical, and physical-psychological (e.g., healthcare personnel psychologically oblige the patient to take the pharmacological treatment) [5]. Mechanical restraint (MR) includes any mechanical device (e.g., the historical straightjacket, the belt with wrist-cuffs, wrist and ankle cuffs tied to the bed, bed-side bars) which, either directly applied to the patient’s body or adjacent to him/her, is hardly removable and aims to prevent, limit, or control his/her body movements. In psychiatry, MR is considered complementary and dependent from manual restraint; the latter is usually employed to implement the former, although it may be used alone as in the case of the forced administration of any medication [6].

Unfortunately, MR still represents one of the greatest concerns in psychiatric clinical practice for each clinician, being considered an obsolete, potentially dangerous, and non-therapeutic approach to treat agitated, aggressive, or violent patients. Restraint, in particular MR, should be used only in cases of severe, not otherwise manageable, emergency situations, when all de-escalation strategies have failed. In Italy, according to the judgment of the Court of Cassation nº 50497, MR has been legitimized to prevent harm to the patient or to others, identifying extreme situations, so called a “state of necessity”, that requires a statement of guardianship from professionals (art.54 and 40 of the Criminal Code, respectively) [7]. Moreover, restraint must be used when unavoidable, as safely as possible, and in a manner that respects the patient’s dignity [8]. The prolonged and inappropriate use of MR, due to its coercive nature, could lead to negative consequences, such as potential permanent damages or death [9]. A dramatic example offered from contemporary chronicle concerns the “Mastrogiovanni’s case”, a patient who died following 84 h of MR [10]. This traumatic event is a very rare phenomenon; however, permanent lesions (of the wrist, legs, nerves), respiratory distress, venous thromboembolism, deep vein thrombosis, especially if the MR is prolonged, could frequently occur. Furthermore, patients report decreased self-esteem and empowerment, strong feelings of humiliation, as well as loss of trust in healthcare personnel [11,12].

The prevalence of MR could vary greatly across different countries, ranging from 0.031 restraint events per day per 1 million in New Zealand to 62.3 restraint events per day per 1 million people in Japan (ages 20–64) [13,14]. Also in Europe, the pattern of coercive psychiatric treatment varies widely between countries with regard to its frequency, type, and legal regulations as well as the rates, duration, and methods of restraint [4,15]. The prevalence of MR in Italy ranges from 3.8% to 20% [16,17,18,19].

Patients with acute symptoms are at the highest risk of being secluded and restrained [20]. Violent behavior or threatening violence is a common indication for the use of seclusion/restraint [21]. Furthermore, above the various psychiatric diagnosis, psychotic symptoms are frequently associated with MR [22].

No-restraint psychiatric wards, called “Club SPDC no-restraint”, deserves mention in Italy, where the use of restraint is strictly forbidden and clinicians use noncoercive methods. Nowadays, only 5% of Italian psychiatric wards are members of the aforementioned type of psychiatric unit [23], while in restraint-using psychiatric wards the common goal is the decrease in mechanical restraint use [17]. This low percentage could be explained as follows: several patients with secondary psychiatric manifestations due to a medical condition, such as major neurocognitive disorders or substances use disorders or delirium, are often admitted to psychiatric units. Another potential explanation is the absence of seclusion in the Italian legislation. Lastly, other related factors involved in the MR phenomenon, including the mental health care system, structure of the psychiatric ward and staff are still undervalued.

Based on the hypothesis that even if restrictive measures in psychiatric clinical practice could be necessary, the prolonged and inappropriate use of MR could lead to negative consequences for the patient and patients’ experiences of restraint could be negative, harmful, or traumatic [24,25], the aim of this study was to estimate the prevalence of MR in an Italian psychiatric unit. Furthermore, a second aim of the present study was to identify the main sociodemographic and clinical characteristics and pharmacological pattern associated with this phenomenon in daily clinical practice to better understand how to minimize the use of MR and organize other specific interventions. The null hypothesis should be the absence of MR in the inpatient psychiatric setting.

## 2. Materials and Methods

### 2.1. Sample

A cross-sectional study has been conducted including all subjects (N = 799) consecutively admitted to the Section of Psychiatry, Department of Neuroscience, Rehabilitation, Ophthalmology, Genetics, Maternal and Child Health (DiNOGMI), University of Genoa (Italy) over three years (2019–2021).

A detailed explanation of the study design was provided and all participants provided a written informed consent for collecting sociodemographic and clinical information, used for research purposes in aggregate mode, anonymously. The study was conducted according to the guidelines of the Declaration of Helsinki [26] and the study design was reviewed and approved by the local ethics committee.

### 2.2. Clinical Assessment

A semi-structured interview, used in a previous published paper [27], was administered to all subjects to collect basic data, as follows: sociodemographic variables (age, gender, marital and occupational status, educational level, living situation, type of discharge, and migrant status), clinical characteristics (primary psychiatric diagnosis, age at onset, duration of illness, non-suicidal self-injuries, first hospitalization, length of current hospitalization in days, long hospitalization (defined as a time over 21 days), type of admission (voluntary or involuntary), need of mechanical restraint, presence of psychiatric and/or medical comorbidity, positive family history of psychiatric disorders). All patients were diagnosed according to the Diagnostic and Statistical Manual for Mental Disorders, fifth edition (DSM-5) [28]. The diagnoses at discharge were divided into the following subgroups: schizophrenia and related disorders, bipolar and related disorders, depressive disorders, personality disorders, and others (including the remaining psychiatric and non-psychiatric disorders such as social admission, mental retardation, or major neurocognitive disorders) [27].

Also, suicidal behaviors (including suicidal ideation, current and lifetime suicide attempt) were considered. Trained psychiatrists assessed suicidal ideation and behavior, according to the definition adopted by Posner et al. [29,30,31] in the Columbia-Suicide Severity Rating Scale (C-SSRS); thus, suicidal ideation included thoughts about a wish to be dead up to active suicide ideation with a specific plan and intent. Furthermore, a suicide attempt was defined as a nonfatal, self-directed, potentially injurious behavior with an intent to die that may or may not have resulted in injury [32,33]. Furthermore, aggressive and violent behaviors were investigated. Direct aggressive behavior is characterized by (a) physical or verbal behavior, often harmful, usually with the intention of inflicting damage or other unpleasantness toward an individual or object, occurring either reactively or without provocation; (b) violent behaviors such as the use of physical force to injure, abuse, damage, or destroy. The World Health Organization (WHO) provides a less conventional definition of violence as follows: “the intentional use of physical force or power, threatened or actual, against oneself, another person, or against a group or community, which either results in or has a high likelihood of resulting in injury, death, psychological harm, maldevelopment, or deprivation”. Lastly, presence of at least one illicit substance (e.g., alcohol, cannabis, cocaine, amphetamines, and heroin) and pharmacological treatment at discharge (e.g., number of medications, antipsychotics, oral or long-acting injection antipsychotics, first or second generation antipsychotics, antidepressants, mood stabilizers, benzodiazepines) were assessed.

### 2.3. Statistical Analysis

The statistical analysis was carried out with the IBM Statistical Package for Social Sciences (SPSS) version 25.0 (SPSS Inc., Chicago, IL, USA) and the value of statistical significance was set at *p* < 0.05. All patients’ characteristics were reported as mean and standard deviation (SD) or frequency and percentage for continuous and categorical variables, respectively. The sample was divided into two subgroups, according to the presence of at least one episode of MR during the index hospitalization in the psychiatric unit. The Kolmogorov–Smirnov test was used to confirm whether all variables in the sample followed a normal distribution. Therefore, the following statistical analyses were performed for the bivariate comparisons: continuous variables were compared using the unpaired Student’s *t*-test for two-class comparisons, categorical variables using the Pearson’s chi-square in contingency tables. Cohen’s d, Cramer’s v, and phi coefficients were reported as measures of effect sizes. Small effect sizes were indicated by d ≥ 0.2 or v|phi ≥ 0.1, medium effect sizes by d ≥ 0.5 or v|phi ≥ 0.3, and large effect sizes by d ≥ 0.8 or v|phi ≥ 0.5.

Finally, a logistic regression analysis was performed using as the dependent variable the MR and including sequentially sociodemographic, clinical, and other characteristics potentially associated with “MR subgroup”.

## 3. Results

The whole sample included 799 patients, of which 14.1% (N = 113) were physically restrained during hospitalization. The mean (±SD) current age was 44.35 (±16.55) and just over half were males (N = 453, 56.7%). About two thirds of patients (N = 523, 65.5%) were single and one out of four (N = 199, 24.9%) were working. One hundred and five (13%) were migrants.

When the two subgroups were compared, the patients who underwent MR were more likely to be male (65.5% vs. 55.2% *p* = 0.042), younger (41.11 ± 17.24 vs. 44.88 ± 16.38, *p* = 0.024), and single (76.1% vs. 63.7%, *p* = 0.020) than patients who did not undergo MR. Finally, MR was more associated with migrant status (22.1% vs. 11.7%, *p* = 0.002). See Table 1 for all characteristics and statistical comparisons.

Mechanical restraint was more prevalent in the patients with other diagnoses (29.2% vs. 17.6%, *p* = 0.006), in patients with aggressive behaviors (83.2% vs. 17.2%, *p* < 0.001), and in patients admitted involuntary (79.6% vs. 20.8%, *p* < 0.001), considering the clinical variables. Furthermore, the status of MR was significantly associated with hospitalization over 21 days (15.9% vs. 9.8%, *p* = 0.049). All statistical comparisons are summarized in Table 2.

Furthermore, the presence of at least one illicit substance was more prevalent among the mechanically restrained patients (53.1% vs. 39.5%, *p* = 0.007), particularly alcohol (38.9% vs. 28.6%, *p* = 0.026) and cannabis (28.3% vs. 15.7%, *p* = 0.001). The other statistical comparisons are summarized in Table 3.

Regarding pharmacological treatment at discharge, patients that underwent MR were taking a lower number of medications (2.66 ± 1.39 vs. 3.21 ± 1.48, *p* < 0.001). Furthermore, a significantly higher prescription of antidepressants (26.7% vs. 8.8%, *p* < 0.001) and lithium (24.1% vs. 14.1%, *p* = 0.020) was in the non-mechanical restrained patients. The other statistical comparisons are displayed in Table 4.

When the logistic regression was performed, aggressive behaviors (OR: 15.124, 95% CI 8.277–27.635, *p* < 0.001), involuntary admissions (OR: 8.662, 95% CI 4.845–15.485, *p* < 0.001), long hospitalization (OR: 3.319, CI 95% 1.498–7.353, *p* = 0.003) and lower number of medications at discharge (OR: 0.800, 95% CI 0.659–0.971, *p* = 0.024) remained significantly associated with the presence of at least one episode of MR in the patients admitted in an emergency psychiatric unit (R^2^ Nagelkerke = 0.523), as shown in Table 5.

## 4. Discussion

Our study aimed to estimate the prevalence of MR in an emergency psychiatric unit and identify the potential sociodemographic and clinical variables empirically associated with the increased use of MR. Nowadays, especially in Italy, it is an extremely debated topic that should be discussed broadly to implement knowledge and understand how limit the use of MR, adopting other operational and clinical strategies or structural environmental changes in the inpatient psychiatric setting [34].

In our sample, the prevalence of MR was 14.1%, in accordance with other Italian studies on this topic [17,18,19].

Being male, younger, single, and a migrant resulted in being significantly associated with at least one episode of MR during the index episode (hospitalization in a psychiatric unit), although these sociodemographic characteristics have been not confirmed with the regression analysis. Our findings are in accordance with data in the literature [35,36,37,38], especially in the Italian context [16,39]. Regarding the association with migrant status, MR could be more used for several reasons including, for example, not only the language barrier, cultural and ethnic differences leading to difficulties in communication (i.e., frequent misunderstandings) between migrants and staff but also different and more severe clinical psychiatric manifestations with poorer insight and pharmacological adherence that needs to be treated [18,40,41].

From the regression analysis, four variables appeared to be independently associated with MR; the strongest variable associated was aggressive behaviors, followed by involuntary admission, long hospitalization, and a lower number of medicaments at discharge.

Aggressive behaviors are quite frequent in the inpatient psychiatric setting and, recently, the number of aggressions has increased. In a systematic review, the weighted mean prevalence of aggressive behaviors was 54% (ranging from 7.5% to 75.9% between studies) [42]. It could be explained by a combination of several factors, differentiated and grouped, as follows: (a) mental health care system factors (regional and hospital policy, ward rules, attitude towards patients, cultural factors); (b) ward factors, including a higher bed occupancy, busy places, overcrowding, day and evening shifts, unsafe and restrictive environment with a lack of structure and inconsistent execution of policy, smoking, a lack of privacy for patients, personal space and freedom to move around, overstimulation, a feeling of physical confinement, inconsistent following of the rules, locked doors without any social interaction; (c) staff factors, including male health care workers, unqualified or more temporary staff, overwork or perceived high workload, job strain or dissatisfaction with leadership, tiredness, lack of good introduction of the nurse, poor collaboration between nurses, burn-out, low grade of communication and de-escalation skills, poor attitudes towards aggression, higher clinician stress level [5,43,44]; (d) patient factors, including a primary diagnosis of a psychotic or bipolar disorder (especially a manic episode) with hostility and impulsivity, involuntary admission due to a lack of insight and the presence of more severe symptomatology but also the loss autonomy and freedom which urges the patient to resist, leading to an aggressive behavior, current illicit substance use, the presence of lifetime aggressive behaviors, younger age, interaction with patients or staff characterized by poor communication, lack of empathy or respect, lack of shared decision making. Also, external stressors are to be considered, including financial worries, having been in foster care as a child, having interpersonal problems, divorced parents or positive first-degree familial psychiatric history, being a migrant [42]. Unfortunately, MR could be the most frequent response to manage an aggressive behavior in psychiatric and non-psychiatric wards, suggesting a lack of skills in managing violence and dangerous situations without any cross-national harmonization in terms of development of best practice recommendations in the field of coercive measures in psychiatry [10]. As a matter of fact, as reported by the EUNOMIA study, the frequency of coercive measures used (i.e., MR) in individual sites showed a high variation across sites [4]. Therefore, it is time for national and international guidelines on MR in psychiatric patients [10].

Recently, de-escalation techniques to manage aggressive behaviors have been provided: always respect the personal space of the patient while maintaining a protected position with the possibility to escape, do not be provoking or offensive, create verbal contact and do not often make eye contact, be concise and communicate clearly, recognize patients’ needs and feelings, pay attention to what the patient is saying without lying to them, agree or agree to disagree with the patient’s thoughts and sensations, lay down the law and set clear and definite confines, propose sincerely the patient’s choices and try to gain their trust, debrief the subject and the staff after the de-escalation [12]. Furthermore, in terms of diagnosis and severe psychopathological conditions, aggressive behaviors are often detectable and MR could be limited and prevented with the use of adequate pharmacological treatment, especially because the first three days are related to the highest prevalence rates of aggressive behaviors [45].

It is also necessary that patients with specific clinical conditions, including substance intoxication or withdrawal, major neurocognitive disorders, social situations, and altered behaviors in neurodevelopment disorders should not be hospitalized in the psychiatric setting. As a matter of fact, in our sample, the “other diagnoses” and presence of at least one illicit substance use (especially alcohol and cannabinoid), were significantly associated with MR contrary to data from the literature [16,37,39,43]. Furthermore, a recent systematic review reported specifically more MR in psychotic disorders (ranging from 26.8% to 82.3%), affective disorders, in particular during a manic episode (varying from 12% to 53.6%), substance use (ranging from 4.9% to 32%), and personality disorders (varying from 1.9% to 11%) [22]. Lastly, a history of aggressive behaviors is considered the most significant risk factor of repeating aggressiveness and, consequently, be treated with MR [46]. It is well-known that these factors are often associated with involuntary admissions that, in our study, resulted in being significantly associated with MR. This could be explained by some predisposing characteristics such as a more severe symptomatology, poor insight, more probability to show specific clinical dimensions such as hostility, impulsivity, aggressiveness, and violence [16,17,39,46,47,48,49], and the association between MR and long hospitalization is quite intuitive. To conclude, health care workers should keep in mind that the several mentioned risk factors are dynamic and interactive in the current inpatient psychiatric setting whereas others are stable and unchangeable, such as previous aggressive behaviors or hospitalization. Therefore, limiting the use of MR is strongly recommended not only for the restriction of freedom and human rights but also for the negative potential consequences, including physical injuries, symptom worsening, a feeling of humiliation, loss of trust in staff, reduced adherence to treatment, decreased self-esteem, permanent lesions, and death due to venous thromboembolism, pulmonary embolism, stress cardiomyopathy, or drug-induced liver injury. It is also necessary to underline that there are situations in which it cannot be avoided [8].

Finally, patients with at least one episode of MR during psychiatric hospitalization were discharged with a lower number of medications. To the best of our knowledge, no studies investigated this significant pharmacological aspect related to MR. This finding could be explained by the fact that the management of aggressive behaviors or severe agitation involves only sedative drugs with a faster action, avoiding the prescription of different overlapping pharmacological classes that induce a polypharmacological effect not only with a higher probability of pharmacological interactions and side effects but also a negative impact on the clinical course of illness [50,51]. It could also depend on the primary psychiatric diagnosis: for example, patients with alcohol intoxication show psychomotor agitation, oppositive behavior and, after de-escalation techniques, MR remains the only potential intervention. Other possibilities are patients affected by psychotic dementia or a major neurocognitive disorder.

The present study has the following limitations. First, the study’s cross-sectional design does not allow for the testing of cause and effect, only providing measures of association. Second, this study was limited to patients admitted to one Italian university hospital (located in the northwestern region of Italy) and the findings could not be generalizable to non-Italian patients, other healthcare systems, or different geopolitical socioeconomic regions. Third, several contributing factors such as those that are staff- and ward-related, that could affect the decision of whether to use MR, have not been considered. Finally, no assessment with structured and validated psychometric tools was made to investigate the potential implicated clinical dimension, such as impulsivity, hostility, and aggressiveness, in the MR phenomenon.

## 5. Conclusions

In our sample, MR is associated with aggressive behaviors, involuntary admissions, long hospitalization, and a lower number of medications at discharge. An early recognition of the patient’s features could improve the clinical handling of the aforementioned conditions and, consequently, decrease the use of MR. It is necessary to work together to limit this phenomenon in the inpatient psychiatric setting, focusing not only on the patient’s characteristics but also on ward- (more green and structured space, privacy and rehabilitation activities, never exceeding the number of beds, no crowding) and staff- (good leadership, training in communication skills, shared decision making) related factors, due to the multifactorial genesis of MR. Future research should focus on longitudinal studies to gain more insight into the dynamics of MR development in the inpatient psychiatric setting, specifically considering these other aforementioned factors that could help identify more precise prevention and intervention strategies.

## Figures and Tables

**Table 1:** Sociodemographic characteristics of the whole sample and the mechanical and non-mechanical restraint subgroups.

N (%) or Mean ± SD	Total Sample (N = 799)	Mechanical Restraint (N = 113)	Non-Mechanical Restraint (N = 686)	Chi^2^/*t*-Test	*p*	Effect Size
Male gender	453 (56.7)	74 (65.5)	379 (55.2)	4.143	0.042	0.072
Current age (years)	44.35 ± 16.55	41.11 ± 17.24	44.88 ± 16.38	2.255	0.024	0.224
Marital status				9.885	0.020	0.111
Single	523 (65.5)	86 (76.1)	437 (63.7)			
Married	132 (16.5)	16 (14.2)	116 (16.9)			
Separated/divorced	121 (15.2)	7 (6.2)	114 (16.6)			
Widowed	19 (2.8)	4 (3.5)	19 (2.8)			
Educational level (years)	11.07 ± 3.60	10.48 ± 3.58	11.17 ± 3.59	1.905	0.057 *	0.192
Occupational status (Yes)	199 (24.9)	24 (21.2)	175 (25.5)	0.946	0.331	0.034
Migrant status	105 (13.1)	25 (22.1)	80 (11.7)	9.303	0.002	0.108
Living situation				0.696	0.706	0.030
Alone	245 (30.7)	31 (27.4)	214 (31.2)			
With parents	464 (58.1)	68 (60.2)	396 (57.7)			
In public residence	90 (11.3)	14 (12.4)	76 (11.1)			
Discharge				0.856	0.652	0.033
Private home	324 (59.0)	64 (56.6)	411 (59.9)			
Transferred to other ward	119 (21.7)	30 (26.5)	155 (22.6)			
Public residence	106 (19.3)	19 (16.9)	120 (17.5)			

* = trend of significance.

**Table 2:** Clinical characteristics of the whole sample and the mechanical and non-mechanical restraint subgroups.

N (%) or Mean ± SD	Total Sample (N = 799)	Mechanical Restraint (N = 113)	Non-Mechanical Restraint (N = 686)	Chi^2^/*t*-Test	*p*	Effect Size
Psychiatric diagnosis Schizophrenia and related disorders Bipolar and related disorder Depressive disorders Personality disorders Others	227 (28.4) 222 (27.8) 80 (10.0) 116 (14.5) 154 (19.3)	35 (31.0) 24 (21.2) 4 (3.5) 17 (15.0) 33 (29.2)	192 (28.0) 198 (28.9) 76 (11.1) 99 (14.4) 121 (17.6)	14.599	0.006	0.135
Age at onset (years)	28.17 ± 14.74	27.16 ± 13.48	28.34 ± 14.94	0.789	0.431	0.082
Duration of illness (years)	16.17 ± 13.40	13.86 ± 12.73	16.56 ± 13.47	1.387	0.198	0.206
Suicidal Ideation	248 (31.0)	32 (28.3)	216 (31.5)	0.455	0.500	0.024
Current suicide attempt	92 (11.5)	7 (6.2)	85 (12.4)	3.656	0.056 *	0.068
Lifetime suicide attempts	136 (17.0)	10 (8.8)	126 (18.4)	6.223	0.013	0.088
Non suicidal self-harm	73 (9.1)	7 (6.2)	66 (9.6)	1.316	0.251	0.041
Aggressive behavior	212 (26.6)	94 (83.2)	118 (17.2)	216.319	<0.001	0.521
Length of hospitalization (days)	10.05 ± 9.58	11.63 ± 12.08	9.79 ± 9.09	−1.898	0.058 *	0.172
Involuntary admission	158 (28.8)	90 (79.6)	143 (20.8)	162.383	<0.001	0.451
First hospitalization	310 (38.8)	51 (45.1)	259 (37.8)	2.224	0.136	0.053
Long hospitalization	85 (10.6)	18 (15.9)	67 (9.8)	3.876	0.049	0.070
Psychiatric comorbidity	379 (47.4)	56 (49.6)	323 (47.1)	0.238	0.626	0.017
Medical comorbidity	313 (39.2)	35 (31.0)	278 (40.5)	3.714	0.054 *	0.068
Family history of psychiatric disorders	326 (40.8)	50 (44.2)	276 (40.2)	0.647	0.421	0.028

* = trend of significance.

**Table 3:** Presence of illicit substances in the whole sample and the mechanical and non-mechanical restraint subgroups.

N (%)	Total Sample (N = 799)	Mechanical Restraint (N = 113)	Non-Mechanical Restraint (N = 686)	Chi^2^/*t*-Test	*p*	Effect Size
At least one substance	331 (41.4)	60 (53.1)	271 (39.5)	7.388	0.007	0.096
Alcohol	240 (30.0)	44 (38.9)	196 (28.6)	4.961	0.026	0.079
Cannabis	140 (17.5)	32 (28.3)	108 (15.7)	10.616	0.001	0.115
Cocaine	96 (12.0)	16 (14.2)	80 (11.7)	0.572	0.449	0.027
Amphetamines	10 (1.3)	1 (0.9)	9 (1.3)	0.143	0.705	0.013
Heroin	47 (5.9)	6 (5.3)	41 (6.0)	0.080	0.777	0.010

**Table 4:** Pharmacological treatment at discharge for the whole sample and the mechanical and non-mechanical restraint subgroups.

N (%) or Mean ± SD	Total Sample (N = 799)	Mechanical Restraint (N = 113)	Non-Mechanical Restraint (N = 686)	Chi^2^/*t*-Test	*p*	Effect Size
Number of medications	3.14 ± 1.48	2.66 ± 1.39	3.21 ± 1.48	3.684	<0.001	0.383
Antipsychotics	637 (79.7)	88 (77.9)	549 (80.0)	0.278	0.598	0.019
Oral	593 (74.2)	76 (67.3)	517 (75.4)	3.333	0.068	0.065
Long-acting injection	104 (13.0)	20 (17.7)	84 (12.2)	2.549	0.110	0.056
First-generation	201 (25.2)	33 (29.2)	168 (24.5)	1.145	0.285	0.038
Second-generation	547 (68.5)	70 (61.9)	477 (69.5)	2.586	0.108	0.057
Antidepressants	193 (24.2)	10 (8.8)	183 (26.7)	16.829	<0.001	0.145
Mood stabilizers	426 (53.3)	55 (48.7)	371 (54.1)	1.140	0.286	0.038
Lithium	181 (22.7)	16 (14.2)	165 (24.1)	5.419	0.020	0.082
Benzodiazepines	563 (70.5)	75 (66.4)	488 (71.1)	1.059	0.304	0.036

**Table 5:** Logistic regression analysis considering sociodemographic and clinical characteristics associated with physical restraint.

	B	S.E.	Wald	*p*	Exp(B)	95% CI for EXP
Male Gender	0.025	0.294	0.007	0.931	1.026	0.576–1.826
Current Age	0.008	0.011	0.581	0.446	1.008	0.987–1.030
Single status	0.417	0.388	1.155	0.282	1.517	0.710–3.243
Migrant status	0.382	0.385	0.985	0.321	1.466	0.689–3.119
Other diagnoses	0.269	0.342	0.619	0.432	1.309	0.669–2.562
Lifetime suicide attempt	0.577	0.455	1.614	0.204	1.782	0.731–4.343
Aggressive behaviors	2.716	0.308	78.006	<0.001	15.124	8.277–27.635
Involuntary admission	2.159	0.296	53.047	<0.001	8.662	4.845–15.485
Long hospitalization	1.200	0.406	8.736	0.003	3.319	1.498–7.353
Presence of illicit drugs	0.212	0.441	0.230	0.632	1.236	0.520–2.935
Alcohol	−0.156	0.417	0.140	0.708	0.855	0.377–1.938
Cannabinoid	0.287	0.404	0.505	0.477	1.332	0.604–2.938
Number of medications	−0.223	0.099	5.074	0.024	0.800	0.659–0.971
Ongoing Lithium treatment	−0.033	0.406	0.007	0.935	0.967	0.436–2.145
Ongoing Antidepressant treatment	−0.208	0.452	0.238	0.625	0.802	0.331–1.944
Constant	−4.789	0.989	23.464	<0.001	0.008	

R^2^ Nagelkerke = 0.523.

## Data Availability

The data presented in this study are available on request from the corresponding author. The data are not publicly available due to privacy/ethical restrictions.
